# HIV care indicators during the COVID-19 pandemic in relation to pre-pandemic care patterns among people with HIV in North Carolina

**DOI:** 10.1080/09540121.2025.2594612

**Published:** 2025-12-04

**Authors:** Courtney N. Maierhofer, Erika Samoff, Brian W. Pence, Abigail N. Turner, Victoria Mobley, John Barnhart, William C. Miller, Kimberly A. Powers

**Affiliations:** a Department of Epidemiology, Gillings School of Global Public Health, University of North Carolina at Chapel Hill, Chapel Hill, NC, USA; b Division of Public Health, North Carolina Department of Health and Human Services, Raleigh, NC, USA; c Division of Infectious Diseases, College of Medicine, The Ohio State University, Columbus, OH, USA; d Division of Epidemiology, College of Public Health, The Ohio State University, Columbus, OH, USA

**Keywords:** HIV care, HIV care disparities, surveillance data, group-based trajectory modeling, SDG3: Good health and well-being

## Abstract

The COVID-19 pandemic abruptly altered the way HIV care was accessed and delivered. We sought to assess HIV care indicators during the COVID-19 pandemic in relation to pre-pandemic HIV care patterns in North Carolina. Using statewide HIV surveillance data and group-based trajectory models, we identified pre-pandemic HIV care trajectories in two partially overlapping populations: (1) newly HIV-diagnosed from March 2014 through February 2018, followed from diagnosis to pandemic start (March 1, 2020); and (2) previously HIV-diagnosed before March 2016, followed from March 2016 to pandemic start. We analyzed pandemic-period HIV care indicators in both populations. In newly diagnosed persons, pre-pandemic HIV care attendance trajectories comprised “consistently high,” “slowly fluctuating,” “steadily decreasing,” and “low U-shaped” groups. Trajectories in previously diagnosed persons were similar, although two distinct low groups replaced the “low U-shaped” group. In both populations, the “consistently high” groups had the highest predicted percentages of persons, while the “low” care groups had the lowest. HIV care indicators in the first pandemic year corresponded with pre-pandemic care patterns: most persons with high and fluctuating pre-pandemic care had an HIV laboratory record in the pandemic year, while most persons in the low care groups had no record in that year.

## Introduction

The COVID-19 pandemic disrupted nearly every facet of daily life, including the delivery of HIV care (Budak et al., 2021; Department of Health and Human Services, 2020; Dolan, 2020; US Department of Health and Human Services, 2021b). At the outset of the pandemic, emergency policies were implemented across the US to reduce SARS-CoV-2 exposure risk in people living with HIV (PLWH), including extensions on days’ supply for antiretroviral therapy (ART) prescriptions, reduced restrictions on telehealth services, and guidance to postpone routine medical and laboratory visits for persons with suppressed HIV viral load and stable health, as feasible (Department of Health and Human Services, 2020; Dolan, 2020; US Department of Health and Human Services, 2021b). Continuous HIV care and ART are necessary to achieve and maintain viral suppression (Cohen et al., 2011; Cohen et al., 2016), and treatment guidelines recommend that PLWH have regular monitoring of HIV RNA levels and CD4 cell counts as markers of treatment reponse (US Department of Health and Human Services, 2021a). The frequency at which these HIV laboratory measures are monitored may vary based on patient-level factors (e.g., ART adherence and viral suppression status) and current guidance from governing health agencies. For example, the World Health Organization (WHO) recommends routine viral load testing at six and 12 months after initiating ART and every 12 months thereafter for stable patients on ART (World Health Organization, 2016), while the US Department of Health and Human Services (DHHS) recommends viral load monitoring every three to four months after ART initiation, with an extension to every six months if a patient is ART-adherent with stable viral suppression for more than one year (Panel on Antiretroviral Guidelines for Adults and Adolescents, 2022; Thompson et al., 2021).

The primary means of monitoring HIV care engagement at local, state, and national levels is with HIV surveillance data (US Department of Health and Human Services. National HIV Surveillance System (NHSS), 2021; Washington Office of National AIDS Policy (US), 2021; Centers for Disease Control and Prevention, 2018), including HIV RNA and CD4 cell count records that are reportable by law in all 50 states (Centers for Disease Control and Prevention, 2021). Given the clinical guidelines recommending laboratory testing at regular intervals (Panel on Antiretroviral Guidelines for Adults and Adolescents, 2022; Thompson et al., 2021; US Department of Health and Human Services, 2021a; World Health Organization, 2016), these records are routinely used as proxies for HIV care visits in estimating HIV care linkage and retention with surveillance data, and are used for the Centers for Disease Control and Prevention’s (CDC) annual HIV reports (Centers for Disease Control and Prevention, 2018; Centers for Disease Control and Prevention, 2020). Directly before the start of the COVID-19 pandemic at year-end 2019, the US had not yet met the National HIV/AIDS Strategy goals for 2020 of having: (1) 90% of PLWH aware of their status, (2) 85% of newly diagnosed persons linked to HIV care within one month of diagnosis, (3) 90% of diagnosed persons retained in HIV medical care, and (4) 80% of diagnosed persons virally suppressed. The CDC estimated that in 2019, 76% of diagnosed PLWH received HIV care (≥1 HIV RNA or CD4 record in a calendar year), and 58% were retained in care (≥2 HIV RNA or CD4 records ≥3 months apart in a calendar year), with considerable variability according to characteristics such as race, ethnicity, and transmission category (Centers for Disease Control and Prevention, 2020). Given the updates to the national HIV goals through the US campaign for Ending the HIV Epidemic (EHE), which aims to achieve at least a 90% reduction in new HIV infections by 2030, understanding population-level HIV care engagement in the context of the COVID-19 pandemic will be vital to its success (Fauci et al., 2019).

In addition to the use of HIV RNA and CD4 records in surveillance data in annual, population-level snapshots of HIV care receipt and retention (Centers for Disease Control and Prevention, 2018; US Department of Health and Human Services, 2021a), these records have also been used to identify and describe longitudinal patterns of HIV care engagement (Enns et al., 2019; Powers et al., 2017), highlighting the dynamic nature of this phenomenon and the potential for important heterogeneities both within and across PLWH over time. In one such study (Powers et al., 2017), trajectory analysis of HIV care retention in persons newly diagnosed with HIV in North Carolina (NC) from 2006 to 2016 identified five distinct longitudinal patterns from the time of HIV diagnosis in three- and six-month intervals, corresponding to “consistently high,” “consistently low,” “steadily declining,” “early increasing,” and “late increasing” care trajectories. Such dynamics have not been widely considered in analyses of COVID-19 pandemic impacts on HIV care patterns. For example, although the CDC reported that population-level HIV care receipt and viral suppression remained fairly stable during the first two years of the pandemic, and that care retention declined in the first year and began to rebound in the second (Centers for Disease Control and Prevention, 2022; Centers for Disease Control and Prevention, 2023), these analyses did not consider potential heterogeneity according to pre-pandemic HIV care history. Additionally, some prior research has assessed the impact of COVID-19 on selected HIV care outcomes (Dawson & Kates, 2020; Santos et al., 2021; Yang et al., 2023), however, these studies have been limited by small sample sizes and short-term follow-up. To assess the extent to which longitudinally defined heterogeneities in HIV care engagement were altered or perpetuated by the COVID-19 pandemic, we used statewide HIV surveillance data in NC to describe key HIV care indicators in the first pandemic year according to pre-pandemic care patterns.

## Materials and methods

### Study design, data source, and populations

We conducted a secondary analysis of PLWH aged ≥13 years using statewide HIV surveillance data in the North Carolina Electronic Disease Surveillance System (NC EDSS) (North Carolina Department of Health and Human Services, 2019a), a web-based communicable diseases surveillance system that contains individual-level information on all HIV diagnoses and HIV RNA and CD4 cell tests performed in NC, as well as demographic, clinical, and behavioral measures. We used these data to identify distinct longitudinal HIV care trajectories before the start of the COVID-19 pandemic, and then to assess HIV care indicators (specified below) in the first year of the pandemic according to these pre-pandemic trajectories. We defined the start of the COVID-19 pandemic in NC as March 1, 2020, based on the first report of a SARS-CoV-2 infection in the state on March 2, 2020, which was followed by the introduction of statewide emergency orders (State of North Carolina, 2022).

**Figure 1.**
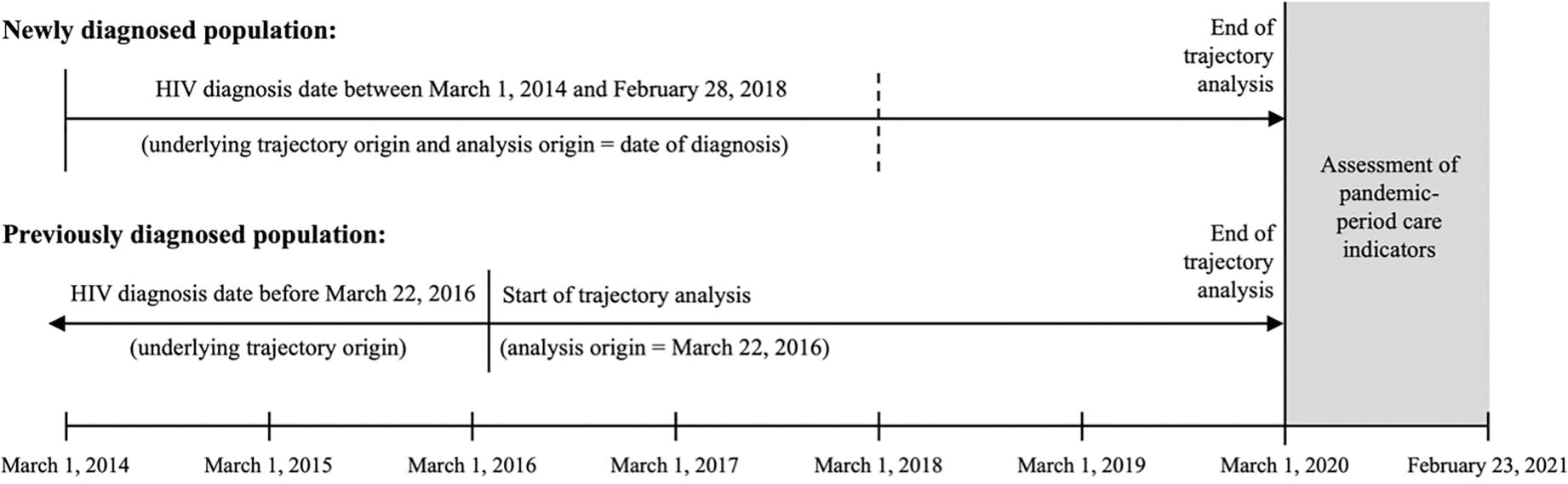
Selection and follow-up for newly and previously HIV-diagnosed trajectory populations.

Recognizing that COVID-19 impacts on HIV care could vary not only according to the type of care trajectory a PLWH was following before the COVID-19 pandemic, but also the point on that trajectory at which the person was situated at the start of the pandemic, we established two primary study populations. For both populations, we conceptualized longitudinal HIV care engagement as a developmental process beginning at HIV diagnosis, with PLWH following different “underlying trajectories” from this “underlying trajectory origin.” To enable assessment of COVID-19 impacts on HIV care among persons who were at different points along these underlying trajectories when the pandemic began, the two study populations were chosen to represent persons who were closer to and further from diagnosis, respectively, in the years leading up to the pandemic (Figure 1). These two populations were not intended to represent mutually exclusive groups but rather to illustrate patterns using two distinct approaches to patient selection and follow-up prior to the start of the COVID-19 pandemic. This design highlights that the timing and method of participant selection may influence the findings for HIV care outcomes during the first year of the pandemic in our analyses.

The first population (hereafter referred to as the *newly diagnosed population*) included persons newly diagnosed with HIV-1 in NC between March 1, 2014 and February 28, 2018. Because North Carolina began requiring the reporting of all HIV RNA and CD4 laboratory tests for surveillance purposes in July 2013, we selected 2014 as the start year for the trajectory analyses to account for potential delays in implementation of the mandate and to ensure more complete HIV laboratory reporting. March of 2014 was selected as the start month to allow persons newly diagnosed with HIV to enter the trajectory cohort for four full years and to be followed for a minimum of 2 years, aligning the trajectory analysis period with the onset of the COVID-19 pandemic in March 2020. This selection process allowed for sufficient follow-up time in all members for trajectory development and identification prior to the start of the pandemic. The origin for pre-pandemic HIV care trajectory analysis in this population was the date of HIV diagnosis (i.e., the “analysis origin” was the same as the underlying trajectory origin).

The second population (hereafter referred to as the *previously diagnosed population*) included all persons diagnosed with HIV-1 in NC before March 22, 2016 who were not known to have died or relocated outside of NC before the start of the COVID-19 pandemic. Persons in the previously diagnosed population who were diagnosed with HIV between March 1, 2014 and March 21, 2016 were also members of the newly diagnosed population, given the overlapping inclusion dates for HIV diagnosis between the first and second population. We selected March 22, 2016 as the origin for HIV care trajectory analysis in the previously diagnosed population to enable identification of care patterns over a period of four full years immediately before the COVID-19 pandemic (i.e., working backwards from March 1, 2020, in 180-day intervals) among persons at relatively later stages of their underlying HIV care trajectories when the pandemic began.

### Trajectory analyses

Separately for each study population, we identified distinct longitudinal trajectories of HIV care engagement from the specified analysis origin over the pre-pandemic period using group-based trajectory models (GBTM) (Nagin, 2005). GBTM is an extension of maximum likelihood methods, known as finite mixture modeling, used to identify clusters of individuals following approximately the same temporal pattern of a given outcome (Nagin, 2005). Based on US guidelines for HIV laboratory monitoring every three to six months, and consistent with both CDC HIV care continuum measures (Centers for Disease Control and Prevention, 2023) and previous studies examining HIV care engagement over time (Crawford et al., 2013; Enns et al., 2019; Health Resources and Services Administration, 2023; Panel on Antiretroviral Guidelines for Adults and Adolescents, 2022; Powers et al., 2017; Thompson et al., 2021), we defined our trajectory outcome of interest as an HIV care visit (yes vs. no) in a given 180-day interval, using documentation of either a CD4 cell count or HIV RNA record as a proxy for a care visit. Using a standard surveillance-based approach to analyzing HIV care engagement (Crawford et al., 2013; Lesko et al., 2015), persons without a CD4 cell count or HIV RNA record during a given 180-day interval were classified as having no evidence of an HIV care visit. That is, missing values within an interval, by definition, indicated the absence of a visit (i.e., the outcome of interest) during that period. We followed persons in each study population from the specified analysis origin through the end of the trajectory follow-up period (February 29, 2020). For the newly diagnosed population, persons who relocated out of NC or died in the first 180-day interval were excluded from analyses, and all others who died or relocated out of NC prior to the end of the trajectory period contributed time up to the interval at which the first of either event occurred.

For each study population, we estimated predictor-free models with PROC TRAJ in SAS v9.4 (SAS Institute Inc, 2013), specifying a logit link and a binomial distribution, for one to five HIV care trajectory groups (Jones et al., 2001; Nagin, 2005; Nagin & Odgers, 2010). We chose the final number of trajectory groups based on interpretability informed by substantive knowledge and the Bayesian Information Criterion (BIC). We assessed the change in BIC value between the less complex model and the more complex model, using a quadratic term for each group, comparing models incrementally in a stepwise fashion from one- to five-group models (e.g., one group vs. two groups, two groups vs. three groups, etc.). After choosing the optimal number of trajectory groups, we selected the shape of each trajectory group using the criteria described above while fitting various polynomial terms (Nagin, 2005; Nagin & Odgers, 2010). In each final model, we ensured that (1) the estimated probability of group membership and the proportion assigned to that group based on the posterior probability of group membership (i.e., the probability that an individual with a specific behavioral profile belongs to a specific trajectory group) were similar, (2) the average of the posterior probabilities of group membership for individuals assigned to each group was ≥0.70, and (3) the confidence intervals around the estimated group membership probabilities were reasonably precise (Nagin, 2005). Additional details on model selection can be found in Supplementary Material.

Within each study population, we calculated the observed overall percentage of persons with an HIV laboratory record in each six-month interval for comparison with the identified trajectories. We also described key characteristics of each HIV care trajectory group using the model-estimated maximum posterior probability of group membership (Nagin, 2005), where each person receives a probability of belonging to a trajectory group and is then assigned to the group with the highest probability. We calculated the number and percentage of persons within categories of each characteristic by assigned trajectory group. Characteristics of interest were race and ethnicity (Black non-Hispanic, White non-Hispanic, Hispanic, Asian or Pacific Islander, American Indian or Alaskan Native, or multiple races), gender (cisgender female, cisgender male, transgender female, transgender male), sex at birth (male or female), age in years at diagnosis (continuous), HIV exposure category [male-male sexual contact, male-female sexual contact, injection drug use (IDU), combined male-male sexual contact and IDU (defined as persons who reported both exposures and were classified separately from those reporting only one), or unknown or other (hemophilia, blood transfusion, perinatal exposure)], and rurality of residential county at diagnosis (urban or rural according to DHHS Office of Rural Health County definitions (North Carolina Department of Health and Human Services, 2019b), where urban is defined as a central county of a metropolitan Core Based Statistical Area (CBSA) with an urbanized core of ≥50,000 residents and surrounding counties with strong commuting integration, and rural is defined as any county that is not a central county of a metropolitan CBSA). Categorical variables were collected as mutually exclusive categories, such that persons could not be classified into more than one group. Additionally, for the previously diagnosed population only, we assessed age in years and number of years from HIV diagnosis (both continuous) at analysis origin. Because gender identity was not captured for any persons diagnosed with HIV prior to 2014, for the previously diagnosed population, we characterized gender using information on gender when available and sex at birth otherwise. We chose this approach rather than multivariable modeling to describe the characteristics of the trajectory groups to avoid challenges of interpretation from multivariable models in the absence of a clear exposure-outcome-confounder relationship of interest (Westreich & Greenland, 2013). More generally, we note that this study was purely descriptive in nature and therefore we did not conduct hypothesis testing in comparing trajectory group characteristics or outcomes.

### Pandemic-period HIV care indicator analysis

In light of the pandemic-related changes to HIV care systems that likely immediately altered the relationship between HIV care and laboratory records (Department of Health and Human Services, 2020; Dolan, 2020; US Department of Health and Human Services, 2021b), we chose to base our assessment of pandemic-period outcomes on simple measures representing minimal levels of HIV care engagement and viral suppression during the first 360 days of the pandemic (March 1, 2020–February 23, 2021). Based on current CDC HIV care continuum measures of care receipt (≥1 HIV RNA or CD4 record) and viral suppression (<200 cp/mL at the most recent HIV RNA record) in a given year (Centers for Disease Control and Prevention, 2018), we classified persons into one of six distinct categories in the 360-day pandemic period: (1) *HIV RNA, most recent suppressed*, defined as having ≥1 HIV RNA record, the most recent of which was <200 cp/mL; (2) *HIV RNA, most recent not suppressed*, defined as having ≥1 HIV RNA record, the most recent of which was ≥200 cp/mL; (3) *CD4 only*, defined as having ≥1 CD4 cell count record and no HIV RNA record; (4) *no HIV lab*, defined as having neither a CD4 cell count nor an HIV RNA record; (5) *relocation out of NC* (address outside of NC any time during the 360-day period); and (6) *death* (death record any time during the 360-day period).

For each study population, we calculated the number and percentage of persons in each HIV care indicator category in the 360-day pandemic period, overall and by pre-pandemic trajectory group (defined according to maximum posterior membership probabilities, as above). For analyses of pandemic-period indicators in the newly diagnosed population, we categorized pre-pandemic relocation and death in two ways: one in which we included persons who died or relocated out of NC during the pre-pandemic period (i.e., all persons included in the pre-pandemic trajectory population), and another in which we excluded all persons who died or relocated out of NC during the pre-pandemic period (i.e., restricted to persons who were alive and living in NC on March 1, 2020).

We performed all statistical analyses using SAS v9.4 (SAS Institute, Cary, NC).

## Results

The newly diagnosed population comprised 4,974 persons diagnosed with HIV in NC between March 2014 and February 2018 (Table 1). The previously diagnosed population comprised 22,218 persons who were diagnosed with HIV before March 22, 2016 and living in NC up to the start of the COVID-19 pandemic. In both populations, most PLWH were male (79.3% newly diagnosed; 71.0% previously diagnosed), Black non-Hispanic (63.2% newly; 63.1% previously), and urban residents (77.6% newly; 76.0% previously). Male-male sexual contact was the primary HIV exposure category among persons with known exposure in both populations (77.8% newly; 63.2% previously); approximately one-third of each population had an unknown HIV exposure type (27.4% newly; 34.1% previously). The median age at HIV diagnosis was 30 years [interquartile range (IQR): 24–44] in the newly diagnosed population and 33 years (IQR: 26–42) in the previously diagnosed population. At the trajectory analysis origin among those previously diagnosed, persons were a median age of 46 years (IQR: 35–54) and had been living with HIV for a median of nine years (IQR: 5–15).

**Figure 2.**
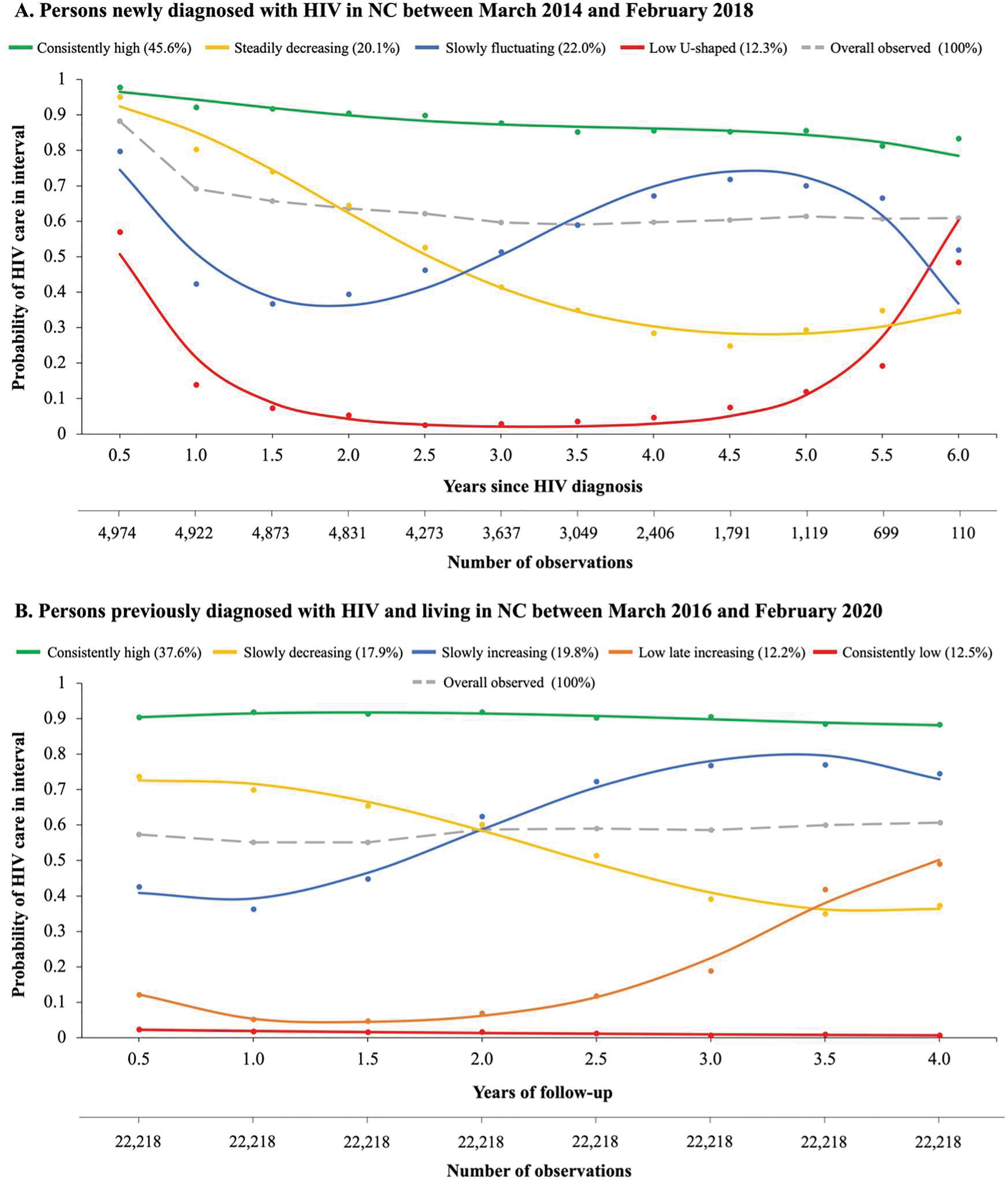
Pre-COVID-19 pandemic longitudinal HIV care trajectories in North Carolina. The solid curves represent the proportion of persons with an HIV laboratory record in a given six-month interval over the study period as estimated by the group-based trajectory model for a given group. The dots represent the observed proportion of persons with an HIV laboratory record in a given interval among those assigned to a given trajectory group based on their maximum posterior group membership probability. The dashed line represents the observed overall proportion of persons with an HIV lab in a given interval. The percentages at the top of each figure represent the predicted percentage of participants following each trajectory. (A) Persons newly diagnosed with HIV between March 2014 and February 2018 were followed from the date of HIV diagnosis through the first occurrence of February 29, 2020, death, or relocation out of state. (B) Persons diagnosed with HIV before March 22, 2016 who were still living in North Carolina at the start of the COVID-19 pandemic (March 1, 2020) were followed from March 22, 2016 through February 29, 2020.

Overall, the observed percentage of newly diagnosed persons with an HIV laboratory record in a given six-month interval dropped from 88% in the first six months after diagnosis to 69% by the end of the first year, remaining relatively steady thereafter in the pre-pandemic trajectory analysis period (Figure 2A). In the previously diagnosed population, the observed percentage of persons with a laboratory record in a given six-month interval was stable at about 60% across the pre-pandemic trajectory analysis period (Figure 2B).

### Pre-pandemic HIV care trajectories

#### Trajectory identification

We identified four latent trajectory groups in the newly diagnosed population over the pre-pandemic period (Figure 2A). The “consistently high” group had the highest predicted percentage of persons (45.6%) and exhibited stable care attendance probabilities exceeding 85% across the trajectory analysis period. The “slowly fluctuating” group had the second-highest predicted percentage of persons (22.0%) and was characterized by an initial drop in the probability of a care visit (from ~80% in the first interval to ~40% in the next three intervals), followed by a slowly increasing probability of HIV care that neared 80% at five years and then began to decline again. The “steadily decreasing” trajectory group comprised a predicted 20.1% of persons, with an estimated 90% receiving care immediately after HIV diagnosis and dropping below 40% after 3.5 years. The “low U-shaped” group had the smallest predicted percentage of persons (12.3%), in whom the estimated probability of care attendance dropped from an initial 51% and remained at or below 20% in years one through five, with a steady increase in the final year of the trajectory analysis period.

We identified five latent trajectory groups in the previously diagnosed population over the four-year trajectory analysis period (Figure 2B). The “consistently high” group had the highest predicted percentage of persons (37.6%) and exhibited stable care attendance probabilities of approximately 90% in each interval. The “slowly increasing” group comprised a predicted 19.8% of the population, with an estimated 40% receiving care immediately after the trajectory analysis origin and an increase to approximately 80% by the end of the trajectory analysis period. The “slowly decreasing” group comprised an estimated 17.9% of the population, in whom the probability of HIV care dropped from an estimated 70% to 40% over the four-year trajectory analysis period. The “low late increasing” and “consistently low” groups had the smallest predicted percentages of persons at 12.2% and 12.5%, respectively. The estimated probability of care in the “low late increasing” group began to rise from approximately 10% after two years to 50% at four years, whereas the “consistently low” group had a near-zero estimated probability of care attendance over the entire trajectory analysis period.

#### Trajectory group characteristics

In the newly diagnosed population, there appeared to be small, but potentially meaningful, differences in the distribution of characteristics across trajectory groups assigned according to maximum posterior membership probabilities (Table 2). The “consistently high” group was older (median 33 vs. 28 years in other groups) and had the highest percentage of White non-Hispanic persons (24.0% vs. ≤ 21.6%) and rural residents (23.9% vs. ≤ 21.7%) compared to other groups. The “low U-shaped” group had the lowest percentage of White non-Hispanic persons (16.9% vs. ≥ 21.5%) and male-male sexual contact (51.0% vs ≥56.5%) compared to the other trajectory groups.

According to maximum posterior probability assignments in the previously diagnosed population, gender appeared to be evenly distributed across trajectory groups, with the exception of a higher percentage of men in the “low late increasing” group compared to the other groups (77.3% vs. ≤ 72.6%) (Table 3). Persons assigned to the “consistently high” group tended to be older at the time of HIV diagnosis (median 36 vs. ≤ 32 years) and at the trajectory analysis origin (median 49 vs. ≤ 45 years). Persons assigned to the “consistently low” group had a higher percentage of Hispanic persons (20.6% vs. ≤ 7.2%) and lower percentages of Black non-Hispanic (57.3% vs. ≥ 61.6%) and White non-Hispanic (18.7% vs. ≥ 23.0%) persons relative to the other trajectory groups. The “consistently low” group had the lowest percentage of male-male sexual contact overall (29.2% vs. ≥ 41.4%); however, HIV exposure category distributions appeared to be similar across trajectory groups when assessed only among those with a known exposure type.

### Pandemic-period HIV care indicators

**Figure 3.**
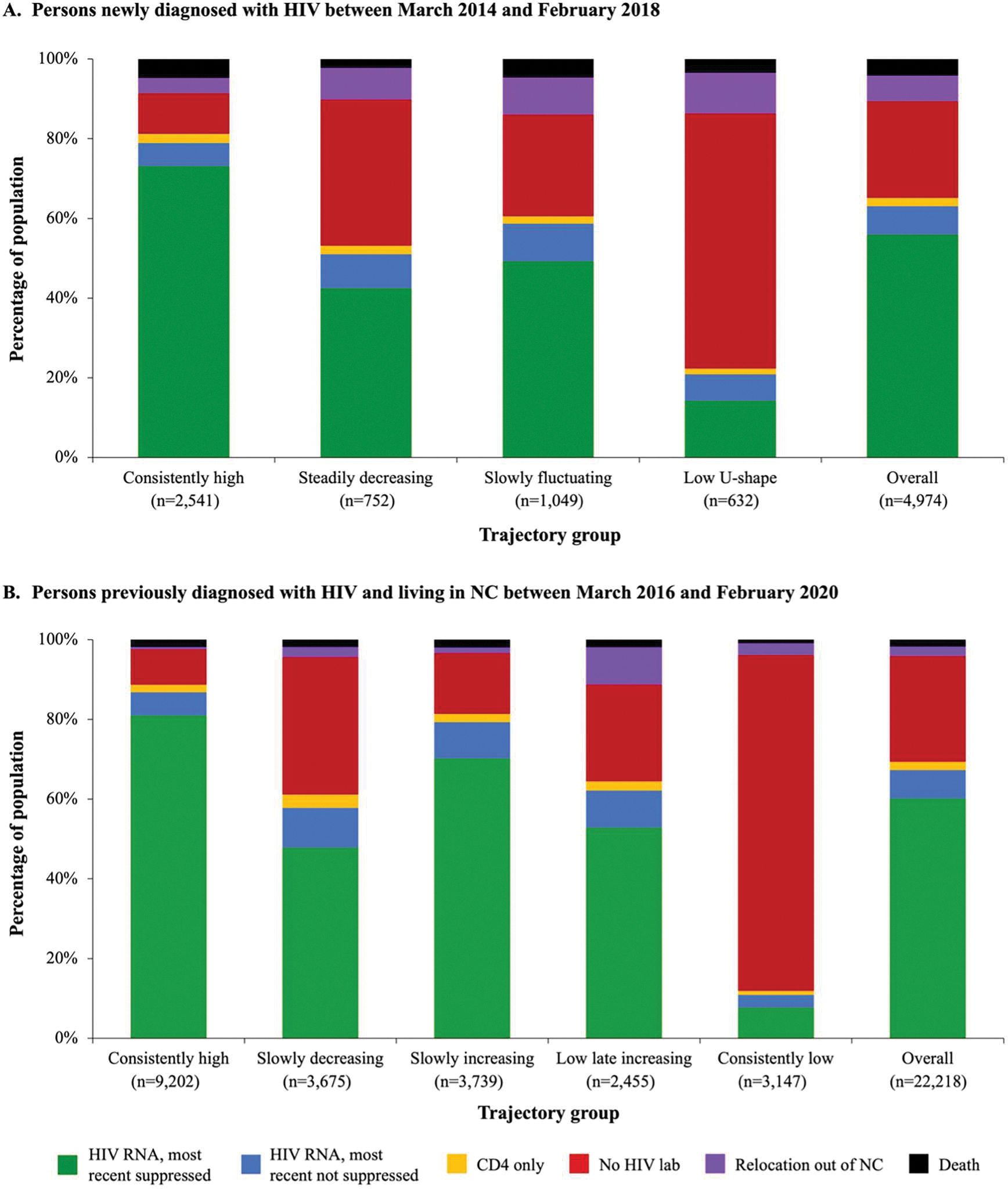
HIV care indicators during the first year of the COVID-19 pandemic according to pre-pandemic HIV care trajectory group. HIV laboratory status was determined for the first 360 days of the COVID-19 pandemic (March 1, 2020–February 23, 2021) overall and among those assigned to a given trajectory group based on their maximum posterior group membership probability. HIV RNA viral suppression was based on last HIV RNA lab recorded in the 360-day period. Note: In panel A, persons who died or relocated out of NC during the pre-pandemic trajectory period are included and categorized according to these pre-pandemic dispositions in the pandemic period totals for each category.

**Figure 4.**
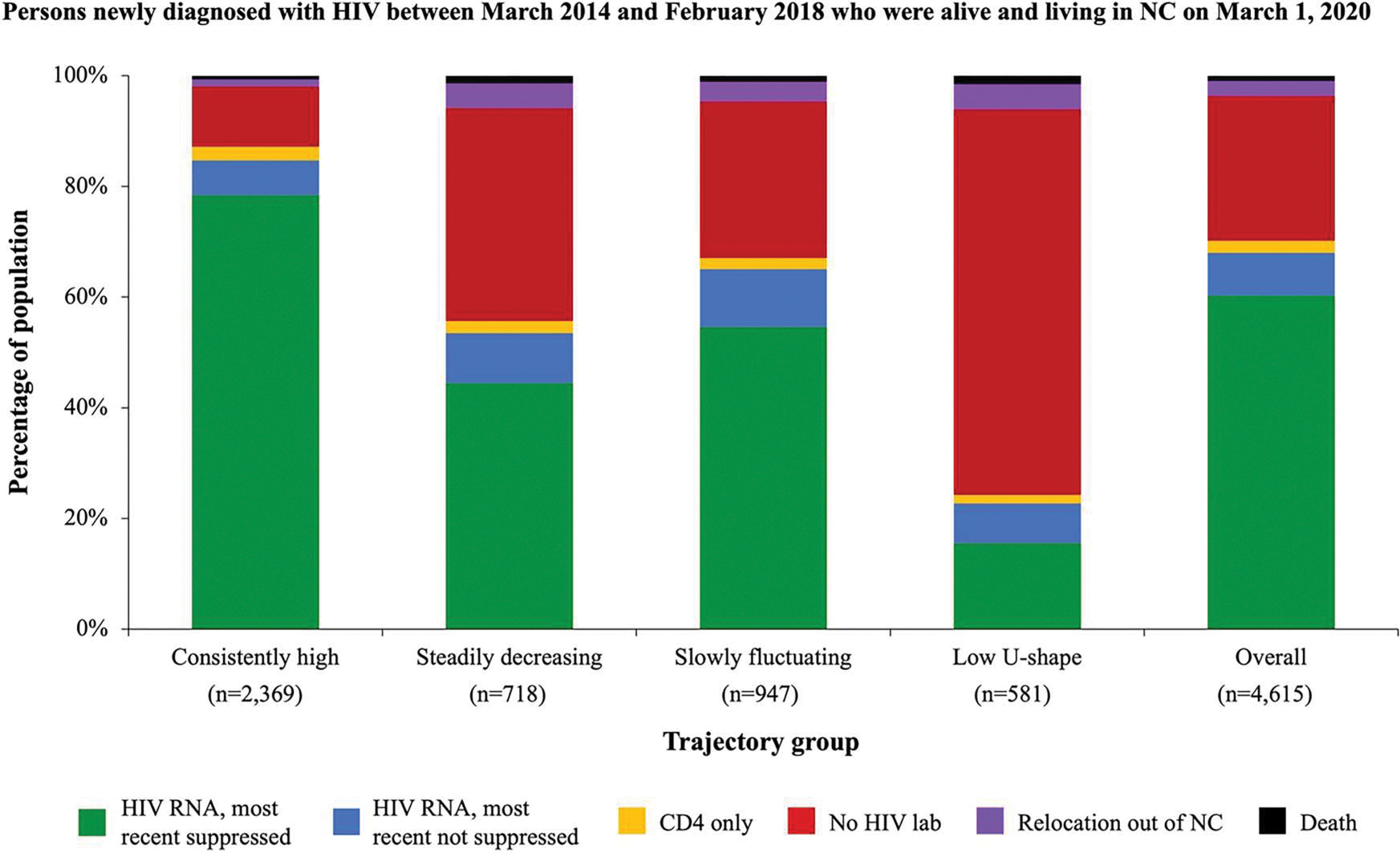
HIV care indicators during the first year of the COVID-19 pandemic according to pre-pandemic HIV care trajectory group among persons newly diagnosed with between March 2014 and February 2018 who were alive and living in NC at the start of the COVID-19 pandemic. HIV laboratory status was determined for the first 360 days of the COVID-19 pandemic (March 1, 2020–February 23, 2021) overall and among those assigned to a given trajectory group based on their maximum posterior group membership probability. HIV RNA viral suppression was based on last HIV RNA lab recorded in the 360-day period. Note: Persons who died (n=165) or relocated out of NC (n=194) during the pre-pandemic trajectory period were excluded from the denominator.

During the 360-day pandemic period between March 1, 2020 and February 23, 2021, 56.0% were virally suppressed and 24.3% had no HIV laboratory record in the newly diagnosed population overall when including all persons from the start of pre-pandemic trajectory follow-up (Figure 3A). According to posterior probability trajectory group assignments, 73.1% of persons in the “consistently high” pre-pandemic HIV care group were virally suppressed and only 10.2% did not have a documented laboratory record in the pandemic period. While 49.3% of the “slowly fluctuating” group was virally suppressed, 25.6% had no laboratory record. In the “steadily decreasing” group, only a slightly higher percentage of persons were virally suppressed (42.4%) than missing an HIV laboratory record (36.7%). Among persons assigned to the “low U-shaped” group, 14.2% were virally suppressed and 64.1% had no HIV laboratory record in the first 360 days of the pandemic. We did not observe any notable differences according to pandemic-period HIV care indicators when excluding persons who died (n = 165) or relocated out of NC (n = 194) during the pre-pandemic trajectory period (Figure 4).

In the previously diagnosed population overall, 60.2% were virally suppressed and 26.6% had no HIV laboratory record in the first year of the pandemic (Figure 3B). Most (81.0%) of those assigned to the “consistently high” group were virally suppressed and only 8.9% did not have an HIV laboratory record in the pandemic period. In the “slowly increasing” group, 70.2% were virally suppressed and 15.3% had no HIV laboratory record in the first year of the pandemic. Approximately half of persons in the “slowly decreasing” and “low late increasing” groups were virally suppressed, while 34.6% and 24.3% had no HIV laboratory record, respectively. Among persons assigned to the “consistently low” group, a large majority (84.3%) had no HIV laboratory record and only 7.7% were virally suppressed at the most recent RNA record over the 360-day pandemic period.

## Discussion

We leveraged seven years of statewide surveillance data, including time before and after the start of the COVID-19 pandemic, to identify distinct longitudinal trajectories of pre-pandemic HIV care in two primary populations of PLWH and describe key HIV care indicators in the first pandemic year according to these prior care patterns. We found that HIV care engagement was dynamic and heterogeneous in the years leading up to the pandemic, and that care outcomes in the first pandemic year were broadly consistent with pre-pandemic patterns.

The HIV care patterns we observed in both analysis populations were similar in shape to those we described in an earlier analysis of HIV care trajectories among persons newly diagnosed with HIV in NC between 2006 and 2016 (Powers et al., 2017). However, our updated analyses suggest a general improvement in HIV care engagement, with a larger predicted percentage of persons in the consistently high care groups in the immediate pre-pandemic years compared to the patterns observed in the decade before 2016, when only 26.2% were predicted to follow a consistently high trajectory on the basis of 6-month intervals. Notably, the predicted percentage with consistently low care in the previous analysis (26.1%) was more than double that in our updated analyses in both populations. These observed improvements in population-level longitudinal HIV care patterns likely reflect National HIV/AIDS Strategy efforts, advancements in HIV treatment, and ongoing improvements in HIV surveillance systems with respect to reporting completeness, data cleaning, and state-to-state collaboration (Antela et al., 2021; Bulstra et al., 2021; Goldstein et al., 2023; U.S. Department of Health and Human Services, 2023).

In both pre-pandemic trajectory populations, more than half of persons overall had an HIV laboratory record in the first year of the pandemic, a majority of whom were virally suppressed. When examining HIV care indicators by trajectory group, we found that the percentage of persons with a laboratory record in the first pandemic year appeared to correspond with pre-pandemic care patterns: most persons with consistently high pre-pandemic HIV care and just over half of those with fluctuating pre-pandemic care had an HIV laboratory record in the pandemic year, while a large majority of those with consistently low and low U-shaped pre-pandemic care had no HIV laboratory record in the first year of the pandemic. These findings are consistent with some other US studies examining HIV care engagement during versus before the pandemic that have reported minimal to no changes in HIV care attendance or viral suppression (El-Nahal et al., 2022; Sorbera et al., 2021), suggestive of a resilient US HIV care system despite incredible pandemic-related challenges to the health care infrastructure overall. However, extending follow-up on HIV care indicators further into the pandemic period in future analyses could provide a better understanding of whether these patterns continued, and how to identify and intervene among persons with decreasing HIV care trajectories prior to the start of the pandemic who appear to have low percentages of post-pandemic HIV viral suppression.

While it has previously been noted that care engagement in some populations of US PLWH was minimally affected by COVID-19 pandemic disruptions (Fadul et al., 2021; Ridgway et al., 2020; San Francisco Department of Public Health, 2020; Tedaldi et al., 2021), HIV care access after the start of the pandemic has been observed to be lower among certain PLWH who had previously experienced heightened challenges to HIV care, including Black and African American persons, Hispanic persons, adolescents, persons who inject drugs, and sexual and gender minorities (Ennis et al., 2022; Gwadz et al., 2021; Kalichman et al., 2020; Kalichman et al., 2021; Sanchez et al., 2020). In both of our analysis populations, persons exhibiting “consistently high” HIV care patterns were older at the time of diagnosis and had higher percentages of White non-Hispanic persons compared to other groups. Previously diagnosed persons in the “consistently low” trajectory group had a strikingly higher percentage of Hispanic persons compared to all other groups, and among the newly diagnosed population, the “low U-shaped” group also comprised a high percentage of persons of color, further reflecting ongoing health disparities.

It is important to note that the latent trajectory groups identified through GBTM are not fixed, directly observable entities. Instead, this approach reduces complex longitudinal patterns into a simpler, interpretable form for guiding public health action. As the final selection of groups was based on both standard statistical measures and more subjective considerations, it is possible that different choices could have resulted in somewhat different findings. Our inclusion of two different analysis populations for pre-pandemic trajectory identification, and the general consistency of pandemic-period findings across those populations, suggests that our overall results may be relatively robust to these types of analytical choices.

We also note that surveillance-based laboratory measures were shown before the COVID-19 pandemic to be acceptable markers of HIV care for monitoring national HIV targets (Centers for Disease Control and Prevention, 2018; Centers for Disease Control and Prevention, 2022; Halperin et al., 2016; Rebeiro et al., 2015), these measurements are not without limitations and may require particularly careful consideration in the context of pandemic-related changes to the care system (Lesko et al., 2015; Miller et al., 2014). For example, because telehealth services were expedited during the pandemic (Budak et al., 2021; Coppock et al., 2021; Smith & Badowski, 2021; Temesgen et al., 2020; US Department of Health and Human Services, 2022), persons who were in fact attending their recommended HIV care visits via telemedicine but did not have corresponding HIV laboratory tests would have been misclassified as “out of care” with surveillance data. Conversely, HIV care retention may have been overestimated in PLWH who were directed to local laboratories for HIV level monitoring without a care visit in order to reduce SARS-CoV-2 exposure. Given the emergency policies related to HIV care implemented during the pandemic, we designed our study to assess HIV care trajectories based on semiannual laboratory measures only up to the start of the pandemic, purposefully limiting our assessment of post-pandemic outcomes to simpler metrics requiring only a single laboratory measure in the first pandemic year. Ongoing assessment of HIV care engagement based on surveillance data may require continued consideration of the correspondence between HIV care attendance and laboratory monitoring going forward.

## Conclusions

While HIV care engagement appeared relatively robust to COVID pandemic disturbances overall, our results highlight long-standing subgroup differences in care engagement that continued into the first year of the COVID pandemic. And although cross-sectional, population-level snapshots of HIV care receipt and retention may remain useful measures of progress toward EHE goals, our analyses reinforce the importance of considering heterogeneities in care engagement across PLWH and over time. As HIV care retention continues to fall short of national goals (Centers for Disease Control and Prevention, 2023; Fauci et al., 2019) and those without continuous HIV care and viral suppression experience the poorest health outcomes (Cohen et al., 2014) and greatest transmission risks (Li et al., 2019), ongoing attention to the dynamic and heterogeneous processes underlying HIV care engagement will be required to end HIV as a public health threat in the US. Understanding these changes within the subpopulations most heavily impacted by the pandemic can help to guide tailored interventions and lay the groundwork for preparedness policies designed to minimize HIV care disruptions in the face of future threats.

## Supplementary Material

Supp 1

Supplemental data for this article can be accessed online at https://doi.org/10.1080/09540121.2025.2594612.

## Figures and Tables

**Table 1.** Baseline characteristics of persons living with HIV in North Carolina by analysis population.

Baseline characteristic^a^	Newly diagnosed with HIV in NC between March 2014 and February 2018		Previously diagnosed with HIV and living in NC from March 22, 2016 through February 29, 2020	
	N = 4,974	(%)	N = 22,218	(%)

**Gender**				
Male	3,945	(79.3)	15,770^c^	(71.0)
Female	979	(19.7)	6,363^c^	(28.6)
Transgender female	48	(1.0)	84^c^	(0.4)
Transgender male	2	(<0.1)	1^c^	(<0.1)
**Race**				
Black, non-Hispanic	3,143	(63.2)	14,019	(63.1)
White, non-Hispanic	1,106	(22.2)	5,232	(23.6)
Hispanic, Latinx	473	(9.5)	1,722	(7.8)
Asian, Pacific Islander	56	(1.1)	112	(0.5)
American Indian, Alaskan Native	30	(0.6)	139	(0.6)
Multiple races	166	(3.3)	992	(4.5)
Missing	0	(0)	2	(<0.1)
**HIV exposure category**				
Male-female sexual contact	562	(11.3)	3,729	(16.8)
Male-male sexual contact	2,807	(56.4)	9,250	(41.6)
Injection drug use (IDU)	112	(2.3)	1,181	(5.3)
Male-male sexual contact, IDU	128	(2.6)	472	(2.1)
Unknown or other	1,365	(27.4)	7,586	(34.1)
**Rurality of residential county**				
Urban	3,861	(77.6)	16,896	(76.0)
Rural	1,113	(22.4)	5,322	(24.0)
	**Median**	**IQR**	**Median**	**IQR**
Age in years at HIV diagnosis^b^	30	(24, 44)	33	(26, 42)
Age in years at baseline	-	-	46	(35, 54)
Years since HIV diagnosis at baseline	-	-	9	(5, 15)

a. At trajectory analysis origin (date of HIV diagnosis in newly diagnosed population and March 22, 2016 in previously diagnosed population)

b. HIV diagnosis date = trajectory analysis origin in newly diagnosed population only

c. Gender identity was not captured for all persons diagnosed before 2014; categorization for n = 2,295 persons without information on gender identity was based on reported sex at birth.

**Table 2.** Baseline characteristics of persons newly diagnosed with HIV in North Carolina between March 1, 2014 and February 28, 2018, by pre-pandemic HIV care trajectory group (N = 4,974).

	Trajectory group							
	Consistently high		Steadily decreasing		Slowly fluctuating		Low U-shaped	
Baseline characteristic^a^	N = 2,541	(%)	N = 752	(%)	N = 1,049	(%)	N = 632	(%)

**Gender**								
Male	1,990	(78.3)	612	(81.4)	832	(79.3)	511	(80.8)
Female	530	(20.9)	133	(17.7)	201	(19.2)	115	(18.2)
Transgender female	20	(0.8)	7	(0.9)	15	(1.4)	6	(1.0)
Transgender male	1	(<0.1)	0	(0)	1	(0.1)	0	(0)
**Race**								
Black, non-Hispanic	1,516	(59.7)	489	(65.0)	705	(67.2)	433	(68.5)
White, non-Hispanic	610	(24.0)	162	(21.5)	227	(21.6)	107	(16.9)
Hispanic, Latinx	268	(10.6)	69	(9.2)	69	(6.6)	67	(10.6)
Asian, Pacific Islander	36	(1.4)	7	(0.9)	7	(0.7)	6	(1.0)
American Indian, Alaskan Native	18	(0.7)	4	(0.5)	6	(0.6)	2	(0.3)
Multiple races	93	(3.7)	21	(2.8)	35	(3.3)	17	(2.7)
**HIV exposure category**								
Male-female sexual contact	314	(12.4)	78	(10.4)	118	(11.3)	52	(8.2)
Male-male sexual contact	1,436	(56.5)	449	(59.7)	600	(57.2)	322	(51.0)
Injection drug use (IDU)	54	(2.1)	17	(2.3)	30	(2.9)	11	(1.7)
Male-male sexual contact, IDU	61	(2.4)	25	(3.3)	32	(3.1)	10	(1.6)
Unknown or other	676	(26.6)	183	(24.3)	269	(25.6)	237	(37.5)
**Rurality of residential county**								
Urban	1,935	(76.2)	589	(78.3)	826	(78.7)	511	(80.8)
Rural	606	(23.9)	163	(21.7)	223	(21.3)	121	(19.2)
	**Median**	**IQR**	**Median**	**IQR**	**Median**	**IQR**	**Median**	**IQR**
**Age in years**	33	(25, 47)	28	(23, 38)	28	(23, 39)	28	(24, 38)

a. Characteristics at trajectory analysis origin (date of HIV diagnosis in this population) among those assigned to a given trajectory group based on their maximum posterior group membership probability.

**Table 3.** Baseline characteristics of persons previously diagnosed with HIV and living in North Carolina between March 22, 2016 and February 29, 2020, by pre-pandemic HIV care trajectory group (N = 22,218).

Baseline characteristic^a^	Trajectory group									
	Consistently high		Slowly decreasing		Slowly increasing		Low late increasing		Consistently low	
	N = 9,202	(%)	N = 3,675	(%)	N = 3,739	(%)	N = 2,455	(%)	N = 3,147	(%)

**Gender** ^b^										
Male	6,416	(69.7)	2,571	(70.0)	2,602	(69.6)	1,898	(77.3)	2,283	(72.6)
Female	2,760	(30.0)	1,090	(29.7)	1,119	(29.9)	542	(22.1)	852	(27.1)
Transgender female	26	(0.3)	14	(0.4)	18	(0.5)	15	(0.6)	11	(0.4)
Transgender male	0	(0)	0	(0)	0	(0)	0	(0)	1	(<0.1)
**Race**										
Black, non-Hispanic	5,811	(63.2)	2,345	(63.8)	2,483	(66.4)	1,578	(61.6)	1,802	(57.3)
White, non-Hispanic	2,301	(25.0)	910	(24.8)	864	(23.1)	570	(23.0)	587	(18.7)
Hispanic, Latinx	551	(6.0)	217	(5.9)	175	(4.7)	132	(7.2)	647	(20.6)
Asian, Pacific Islander	51	(0.6)	20	(0.5)	15	(0.4)	7	(0.2)	19	(0.6)
American Indian, Alaskan Native	62	(0.7)	31	(0.8)	25	(0.7)	5	(0.2)	16	(0.5)
Multiple races	426	(4.6)	152	(4.1)	177	(4.7)	163	(7.8)	74	(2.4)
Missing	0	(0)	0	(0)	0	(0)	0	(0)	2	(<0.1)
**HIV exposure category**										
Male-female sexual contact	1,604	(17.4)	625	(17.0)	630	(16.5)	413	(16.8)	457	(13.8)
Male-male sexual contact	3,811	(41.4)	1,640	(44.6)	1,587	(42.1)	1,232	(50.2)	980	(29.2)
Injection drug use (IDU)	491	(5.3)	189	(5.4)	196	(5.7)	141	(5.7)	164	(6.8)
Male-male sexual contact, IDU	167	(1.8)	77	(2.7)	70	(2.4)	99	(4.0)	59	(2.2)
Unknown or other	3,129	(34.0)	1,144	(31.1)	1,256	(33.6)	570	(23.2)	1,487	(47.3)
**Rurality of residential county**										
Urban	6,918	(75.2)	2,771	(75.4)	2,915	(78.0)	1,869	(76.1)	2,423	(77.0)
Rural	2,284	(24.8)	904	(24.6)	824	(22.0)	586	(23.9)	724	(23.0)
	**Median**	**IQR**	**Median**	**IQR**	**Median**	**IQR**	**Median**	**IQR**	**Median**	**IQR**
**Age in years at HIV diagnosis**	36	(27, 44)	32	(25, 41)	32	(25, 41)	31	(24, 40)	32	(25, 40)
**Age in years at baseline**	49	(38, 56)	44	(32, 53)	45	(35, 53)	43	(33, 52)	45	(36, 53)
**Years since diagnosis at baseline**	10	(5, 16)	8	(4, 14)	9	(5, 15)	9	(5, 15)	11	(6, 16)

a. Characteristics at trajectory analysis origin (March 22, 2016 in this population) among those assigned to a given trajectory group based on their maximum posterior group membership probability.

b. Gender identity was not captured for any persons diagnosed before 2014; n = 2,295 persons without information on gender identity were reported as sex at birth (n = 14 slowly decreasing; n = 15 slowly increasing; n = 458 low late increasing; n = 1,808 consistently low).
