# Supplementary material for: HIV care indicators during the COVID-19 pandemic in relation to pre-pandemic care patterns among people with HIV in North Carolina
Source: AIDS Care. Author manuscript; Available in PMC 2026 Feb 1. (PMC12860448; doi:10.1080/09540121.2025.2594612)
Supplement: Supp 1 [file NIHMS2134569-supplement-Supp_1.docx]

**SUPPLEMENTARY MATERIAL**

HIV care indicators during the COVID-19 pandemic in relation to pre-pandemic care patterns among people with HIV in North Carolina

For model selection in each study population, we separately and sequentially fit one- to five-group predictor-free models with PROC TRAJ in SAS v9.4, using a logit link, binomial distribution, and quadratic term for each group (**Supplementary Table 1**). We decided a priori to fit a maximum of five groups to ensure interpretability of our results. To determine the optimal number of trajectory groups we used the following criteria: 1) change in Bayesian Information Criterion (BIC) by subtracting the BIC value of the less complex model from the BIC value of the more complex model, comparing models in a step-wise manner (one vs. two groups, two vs. three groups, etc.), 2) the estimated probability of group membership and the proportion assigned to that group based on the posterior probability of group membership (i.e., the probability that an individual with a specific behavioral profile belongs to a specific trajectory group) were similar, 3) the average of the posterior probabilities of group membership for individuals assigned to each group was ≥0.70, 4) group size was adequate, where each group included ≥5% of the sample, and 5) confidence intervals around the estimated group membership probabilities were reasonably precise, and 6) the principle of parsimony.^26^

After choosing the optimal number of groups for each study population, we then selected the shape of each trajectory group using both BIC values and the substantive knowledge described above, fitting varying combinations of polynomial terms. In persons newly diagnosed with HIV, the five-group model produced the highest BIC values in terms of number of participants and number of observations. Based on BIC values and precision of estimates, the best-fitting model was a four-group model using two quadratic and two cubic terms (2, 2, 3, 3), with a final subject level BIC of -19599.80 and a final observation level BIC of -19616.80. In persons living with HIV, the five-group model produced the highest BIC values in terms of number of participants and number of observations. The best-fitting five-group model was modeled using a linear term, and four cubic terms (1, 3, 3, 3, 3), with a final subject level BIC of -95895.70 and a final observation level BIC of -95918.57.

**Supplementary Table 1. BIC values for the selection of each trajectory model with the optimal number of groups by varying number of group trajectories using all quadratic terms**

| **Model** | **BIC values** | | | | |
| --- | --- | --- | --- | --- | --- |
|  | **Newly diagnosed with HIV between March 2014 and February 2018** | |  | **Living with HIV from March 22, 2016 through February 29, 2020** | |
|  | **Observation level**  **N=36,764** | **Subject level**  **N=4,974** |  | **Observation level**  **N=177,744** | **Subject level**  **N=22,218** |
| One-group | -22856.02 | -22853.02 |  | -120745.30 | -120742.20 |
| Two-group | -20103.02 | -20096.02 |  | -99268.09 | -99260.81 |
| Three-group | -19798.21 | -19787.21 |  | -96940.45 | -96929.02 |
| Four-group | -19702.26 | -19687.25 |  | -96281.25 | -96265.66 |
| Five-group | -19643.40 | -19624.39 |  | -96048.14 | -96028.38 |
